# Floating aortic thrombus with celiac artery embolus presenting with chronic epigastric and right upper quadrant pain: A case report

**DOI:** 10.1002/ccr3.3791

**Published:** 2021-01-12

**Authors:** Fatemeh Jahanshahi, Alireza Kalantar Motamedi

**Affiliations:** ^1^ Student Research Committee, Faculty of Medicine Iran University of Medical Sciences Tehran Iran; ^2^ Department of Vascular Surgery, Rasool Akram Medical Complex Iran University of Medical Sciences Tehran Iran

**Keywords:** arterial obstruction, chronic pain, Floating aortic thrombus

## Abstract

Arterial occlusion may be the cause of chronic pain, and vascular diagnostic procedures should be a part of the workup in patients with unexplained chronic visceral pain.

## Graphical abstract

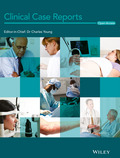

## 1 INTRODUCTION

Our case was a 74‐year‐old woman with chronic abdominal pain in the epigastric and right upper quadrant region, may be caused by an embolic event involving the celiac artery. The source of the embolus is believed to be from a floating aortic thrombus, involving the descending thoracic aorta.

Floating aortic thrombus is relatively uncommon and is a mostly asymptomatic occurrence, and if embolic events do not occur, they can be completely silent. Floating thrombus often occurs in the atherosclerotic or aneurysmal aortic wall, and/or at their dissection sites or on an ulcerated atheroma. In other words, floating thrombus of the aorta does not occur primarily within a normal aorta most of the time.^1^, ^2^

Risk factors such as hypercoagulable states, smoking, steroid use, and oral contraceptive use can make an individual prone to thrombus formation. The most common site of thrombus formation is the descending aorta with a probability of 37.5%.^3^, ^4^

The most prevalent sign in these patients is embolic events, which can cause ischemia of the extremities, visceral Ischemia, and/or a critical situation requiring emergent surgical intervention. Chronic pain and chronic ischemia resulting from embolic events are rarely mentioned in reported cases. Therefore, embolic phenomena may be overlooked as the etiology of the pain in these cases.

Owing to the morbidity and mortality occurred following the surgical intervention in these patients, more conservative treatments are highly welcomed. However, there are still many cases that show an unsatisfactory response to this type of treatment. Recently, thoracic endovascular aortic repair (TEVAR) has become one of the proposed methods for managing these patients.^4^

In this article, a patient with several months of chronic abdominal pain is reported. In this case, a floating thrombus in the descending aorta was the root cause of the embolic event that inflicted the pain, which was managed by TEVAR and adjuvant therapy with an anticoagulant drug.

## 2 CASE REPORT

The patient was a 74‐year‐old woman with a history of chronic abdominal pain in the last several months. During this period, the pain was intermittent, and its severity fluctuated regardless of eating. The pain often radiated to the right upper quadrant region. Additionally, in the last 20 days, she reported watery diarrhea and occasional vomiting. Colonoscopy and upper gastrointestinal (GI) endoscopy performed by a gastroenterologist were unremarkable.

The patient had a history of cholecystectomy conducted two years ago, and she had a history of chronic hypertension (HTN) for which she had been using losartan to control chronic hypertension (HTN). The examination revealed a soft abdomen with no guarding or distention. Mild tenderness was reported in the epigastric area and right upper quadrant (RUQ) as well as increasing the bowel sounds, and no organomegaly was reported on deep palpation.

The patient's vital signs were pulse rate 87/ min, temperature 37.2°C, blood pressure 130/70 mm Hg, and respiratory rate 17 breaths/min.

Abdominal and pelvic ultrasonography was requested and the results revealed mild dilation of the common hepatic duct that was considered normal regarding her history of cholecystectomy. Magnetic resonance cholangiopancreatography (MRCP) was done that revealed a top normal size common bile duct. The results of laboratory tests showed alkaline phosphatase = 256 U/L (upper 306), AST = 30 U/L, ALT = 30 U/L (normal up to 31), BUN = 7 mg/dL, Cr = 0.6 mg/dL, and normal calcium, phosphorus, sodium, and potassium levels. MRCP was conducted that its report showed the common bile duct which was top normal in diameter (9.5‐10 mm in the proximal portion and 8.5 mm in the distal portion) and no intraluminal filling defect was noted within the common bile duct. Apparent narrowing in the distal end of the CBD was observed.

Stool examination showed brown watery stool with 1‐2 WBC and 0‐1 RBC with 1+ occult blood and no ova, parasites, or protozoa. Stool culture showed growth of normal stool flora.

The four‐day follow‐up tests showed AST = 53 U/L (normal up to 31), ALT = 35 U/L (normal up to 31), alkaline phosphatase = 314 U/L (normal up to 306), amylase 23 U/L (up to 100), lipase = 26 U/L (up to 60), total bill = 1.2 mg/dl (upper 1.2), direct bill = 0.3 mg/dL (up to 0.3), and gamma‐glutamyl transferase (GT)=29 U/L (up to 32). These findings highlighted nonsignificantly elevated liver enzyme levels.

**FIGURE 1:**
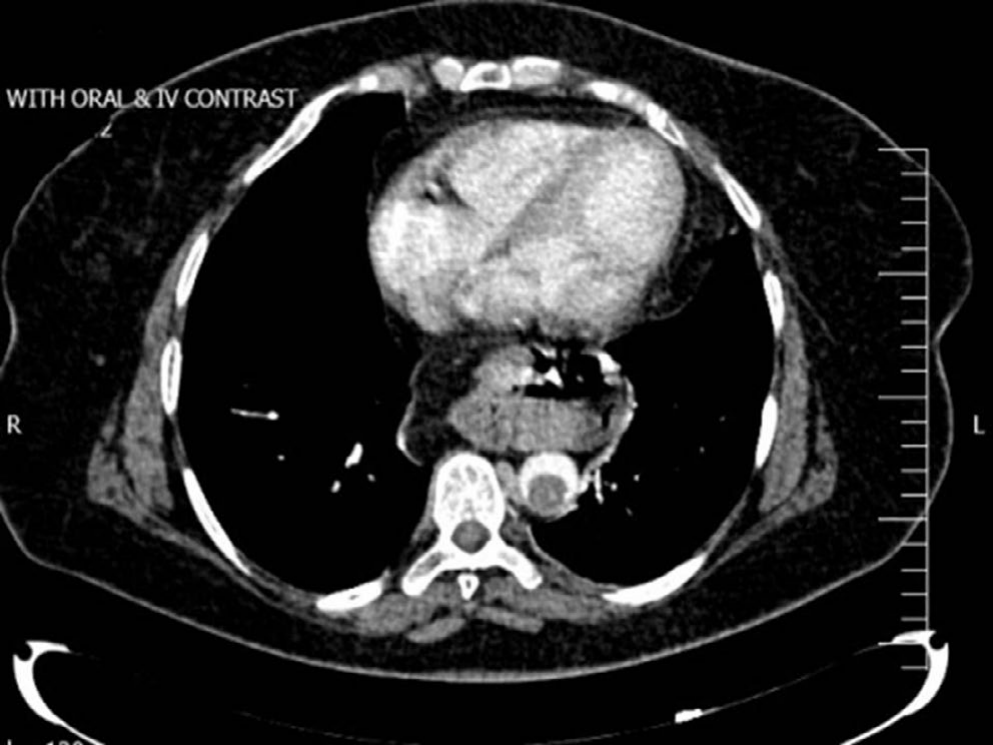
Abdominopelvic CT scan with oral and IV contrast, axial view: a floating thrombus in aortic artery lumen with transverse diameter of 16 mm

**FIGURE 2:**
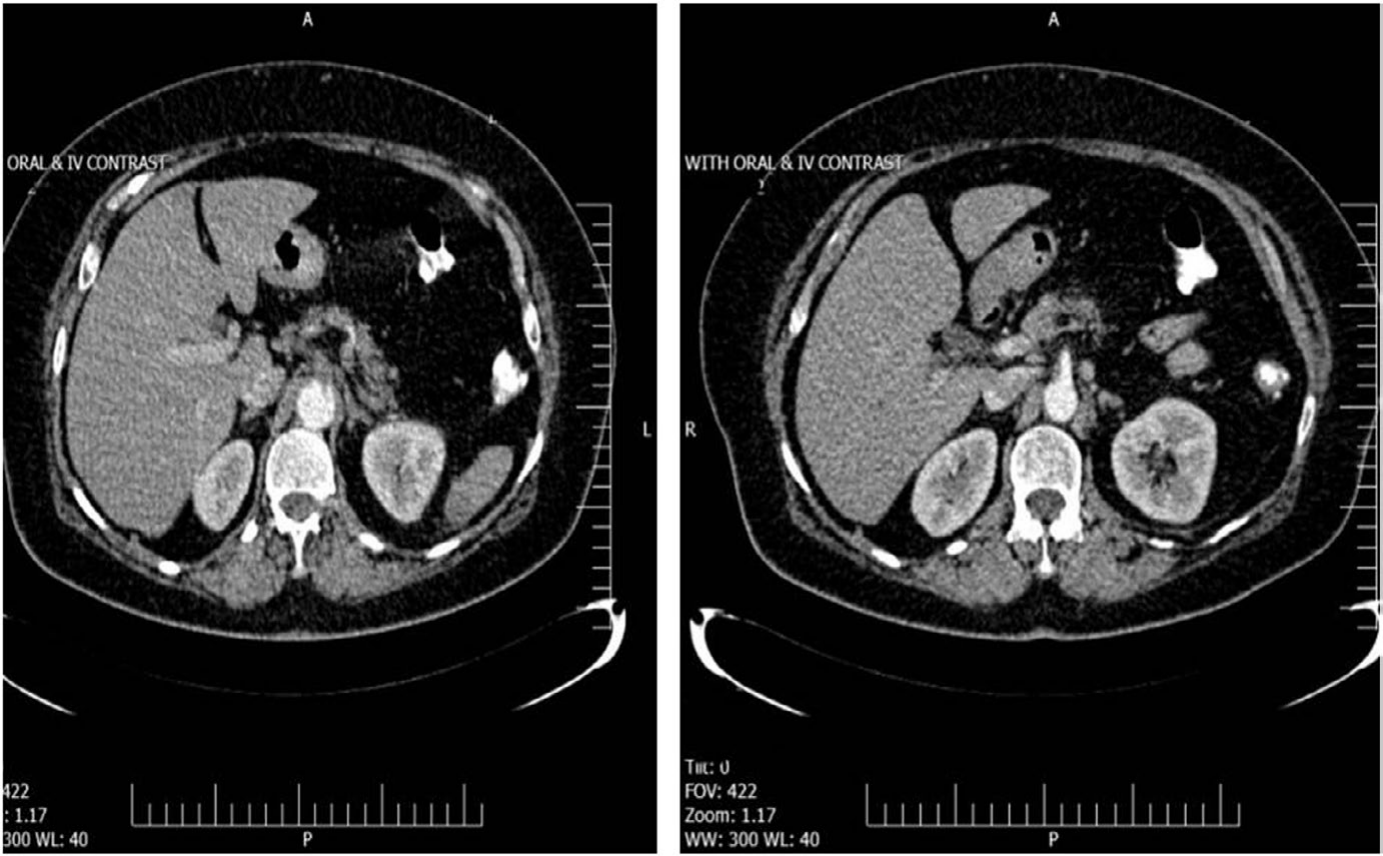
A Thromboembolus in origin of celiac trunk

Finally, abdominopelvic computed tomography (CT) with and without intravenous contrast was requested, which demonstrated a focal intraluminal fusiform pedunculated filling defect protruding from the posterior wall of the distal thoracic aorta into the lumen with 34 mm in length and 16 and 17 mm transverse and anteroposterior diameters, respectively, suggestive of focal mural thrombus (Figure 1). Moreover, a thromboembolus was observed in the origin of the celiac artery (Figure 2).

The patient underwent thoracic endovascular aortic stenting, and an aortic stent graft with appropriate size was used to cover the thrombus of the descending thoracic aorta. However, no further treatment was undertaken concerning the celiac artery occlusion, other than to manage it with an anticoagulant drug, because of the chronic nature of the celiac embolus—which not leading to bowel ischemia or gangrene due to the presence of multiple collateral vessels.

The postoperative course was uneventful, and the patient was discharged the following day. One month later in the postoperative follow‐up visit, the patient stated that the abdominal pain was eliminated and she no longer suffered from diarrhea and vomiting any more. In 2 years of follow‐up, no evidence of recurrent embolic events was noted.

## 3 DISCUSSION

Floating thrombus in the aorta is a rare finding and often secondary to atherosclerotic plaques, endothelial arterial disorders, aortic dissection, and aneurysms. In fact, the primary formation of floating thrombus is relatively few and the pathway is not established yet.^5^

In a study by Machleder et al, the incidence of aortic thrombosis in 10 671 autopsy cases understudy was reported to be approximately 0.9%.^6^

Although the exact pathology of thrombus formation in the aorta is not completely determined, several risk factors and predisposing factors have been suggested in the literature. Risk factors include senility, obesity and BMI > 30 kg/m^2^, diabetes, dyslipidemia, any condition that makes someone prone to atherosclerosis, hypertension, smoking, hormone replacement therapy, and any underlying disease that motivates thrombus formation including thrombocythemia or lack of proteins C and S, antiphospholipid syndrome, and malignancies.^2^, ^3^, ^4^

A floating thrombus in the aorta can be completely asymptomatic and unrecognizable, though it is a potential threat that can lead to dramatic embolic events. Depending on thrombus location, embolic events may lead to stroke and appear with neurologic signs, or cause occlusion of upper extremity vessels and upper limb ischemia. The embolus can cause visceral ischemia and Infarct, which manifest with severe abdominal pain, or they may obstruct the iliac, femoral, or popliteal vessels with symptoms such as claudication, ischemic ulcers, and gangrenous limbs.^6^

Reports on emboli in visceral arteries often indicate the involvement of renal, celiac, or mesenteric arteries. Reduced or absent blood flow causes ischemia and kidney gangrene in the case of renal arteries, spleen ischemia and infarct in the case of the splenic artery, and mesenteric ischemia and gangrene if the superior mesenteric artery is involved. However, occlusion in arteries such as the hypogastric and celiac may not cause severe acute pain, and instead, the patient enters the process of chronic visceral ischemia and chronic pain that rarely provokes suspicion of missed arterial emboli. As it was the case in this report, since the celiac trunk is not a major provider of blood supply to the bowel mesentery and collateral blood supply is abundant, we assume that this occlusion caused mild chronic bowel ischemia, with mild epigastric and right upper quadrant pain in our patient which was gradually compensated by collateral vessels.^5^

Transesophageal echocardiography (TEE) is suggested as the most useful diagnostic modality for determining the accurate site of thrombi inside the aorta. Moreover, it allows higher accuracy and sensitivity for differential diagnosis of other aortic diseases. However, it suffers some limitations in surveying the ascending aorta and the upper portion of the descending aorta which are distant from the trachea. Aside from TEE, CT angiography and MR angiography are also applicable to diagnosis. Angiography, despite providing more accurate results, is often used in situations in which intravascular therapeutic interventions are also considered since it is an invasive approach.

It is worth mentioning that with advances in diagnostic modalities, although aortic thrombus and embolic events are rare, many cases have been identified and reported in the literature. In each of these reported cases, a specific treatment methodology has been applied. However, to date, no particular standard of treatment protocol has been established for managing these patients, and the treatment approach is mostly based on the patient's condition and the physician‘s expertise.

It is, therefore, obvious that various treatment methodologies for these patients present different levels of efficacy and invasiveness. Treatment methodologies can be categorized into conservative treatments, including using anticoagulants and thrombolytic agents; less‐invasive interventions such as thromboaspiration, balloon catheter thrombectomy, and endovascular stent grafts; and invasive treatments, including surgical thrombectomy and thromboembolectomy, and aortic replacement.^7^

Generally, the prognosis of these patients is not superb and they might experience several embolic events further throughout their lifetime. Nevertheless, according to the literature, the number of patients who live afterward with no complication is not few.^8^

## 4 CONCLUSION

Acute arterial obstruction commonly presents with acute‐onset pain and ischemia. However, as it was the case in this report, the arterial obstruction may be the cause of chronic pain, and vascular diagnostic procedures should be a part of the workup in patients with unexplained chronic visceral pain.

## 5 CONSENT FOR PUBLICATION

Written informed consent was obtained from the patient for publication of this case report. A copy of the written consent is available for review from the corresponding author on request.

## CONFLICT OF INTEREST

There is no conflict of interest to declare.

## AUTHOR CONTRIBUTIONS

FJ: conceived and designed the study. AKM: acquired the data. FJ: drafted the manuscript. FJ and AKM: critically revised the manuscript for important intellectual content. AKM: supervised the study. All authors read and approved the final manuscript.

## ETHICAL APPROVAL

Institutional review board approval for case report is not required at our institution. To keeping ethical principles, name of the patient was not pointed in the paper and the rights of the subject were protected. The patient received treatment consistent with the current standard of care.

## Data Availability

The authors agree with sharing, coping the data used in this article, even for commercial purposes. The data of this study are available from the corresponding author on reasonable request.
